# The Use of DeepQSAR Models for the Discovery of Peptides With Enhanced Antimicrobial and Antibiofilm Potential

**DOI:** 10.1002/minf.70029

**Published:** 2026-04-16

**Authors:** Jiaying You, Hazem Mslati, Evan F. Haney, Noushin Akhoundsadegh, Robert E. W. Hancock, Artem Cherkasov

**Affiliations:** ^1^ Vancouver Prostate Centre Department of Urologic Sciences Faculty of Medicine University of British Columbia Vancouver BC Canada; ^2^ Department of Cellular and Molecular Medicine Faculty of Medicine University of Ottawa Ottawa ON Canada; ^3^ Centre for Microbial Diseases and Immunity Research Department of Microbiology and Immunology Faculty of Science University of British Columbia Vancouver BC Canada

## Abstract

Increasing concerns regarding prolonged antibiotic usage have spurred the search for alternative treatments. Antimicrobial peptides (AMPs), first discovered in the 1980s, have exhibited significant potential against a broad range of bacteria. Short‐sequenced AMPs are abundant in nature and present across various organisms. Recently, machine learning technologies such as Quantitative Structure Activity Relationships (QSAR) have enabled expedited discovery of potential AMPs with broad‐spectrum antibacterial activity as the amount of available AMP training data increases. Among those, Deep QSAR has recently emerged as a distinct type of application that utilizes conventional molecular descriptors in conjunction with more powerful deep learning (DL) models. Here, we demonstrate the power of Deep QSAR in predicting broad‐spectrum AMP activity. Using a recurrent neural network–based QSAR model, we achieved nearly 90% fivefold cross‐validated accuracy in classifying AMP activity. Using the developed approach, we designed 98 novel peptides, of which 36 experimentally demonstrated more effective antibiofilm activity and 26 peptides exhibited stronger antimicrobial activity compared to a well‐characterized host defense peptide IDR‐1018, which was demonstrated to possess broad spectrum antibiofilm activity against a wide range of bacterial pathogens and a previous computer‐aided peptide design study employing IDR‐1018 derivatives successfully identified novel peptides with enhanced antibiofilm activity. Notably, 22 of those peptides demonstrated improvements of both antimicrobial and, particularly, antibiofilm properties, making them suitable prototypes for preclinical development and demonstrating efficacy of DeepQSAR modeling in identifying novel and potent AMPs.

## Graphical abstract

A DeepQSAR framework integrating a feed‐forward neural network and a recurrent neural network (RNN) was developed to identify antimicrobial peptides with enhanced antibiofilm and antimicrobial activity. The feed‐forward model was trained on in‐house antibiofilm IC_50_ data to capture quantitative structure–activity relationships, and its learned features were transferred to an RNN classifier trained on large public AMP datasets. This combined transfer‐learning approach enabled simultaneous prediction of biofilm inhibition and antimicrobial potential. Approximately 50,000 peptide fragments derived from human proteins were screened in silico, clustered for diversity, and top candidates were synthesized for experimental validation. Multiple peptides demonstrated superior activity compared to the reference peptide IDR‐1018, particularly against MRSA biofilms. The results highlight the power of DeepQSAR‐driven peptide design for accelerating discovery of multifunctional antimicrobial agents.

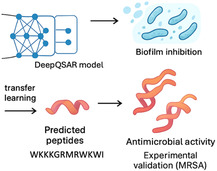

## 1 Introduction

Antibiotic pollution is becoming a global environmental challenge, driven by everyday human activities [1, 2, 3, 4, 5, 6, 7, 8]such as livestock farming, agriculture, waste disposal, and urban wastewater discharge [9]. The inappropriate disposal of antibiotics to the air, soil, and water has forced living organisms to develop defensive mechanisms, namely antimicrobial resistance (AMR) [10], weakening the effectiveness of modern medicine in treating infections. As a result, healthcare costs have risen, as additional treatments and interventions become necessary to combat resistant infections.

One promising alternative to the conventional use of small molecule antibiotics is to utilize antimicrobial peptides (AMPs). These naturally occurring substances are found in nearly all living organisms and serve as a crucial component of the immune system, providing broad‐spectrum protection against bacteria, fungi, parasites, and viruses and biofilms and modulating immunity. Notably, AMPs exhibit unique mechanisms of potent, selective, and direct bacterial destruction resulting in a lower propensity for pathogens to develop resistance [11, 12].

Overall, the growing concern regarding AMR has driven intensive discovery and design of novel AMPs with enhanced efficacy and stability. In recent years, advances in computational tools [13] have significantly accelerated the process in identifying novel potent peptides. For example, machine learning (ML) [14, 15, 16] and deep learning (DL) [17, 18, 19] models have been increasingly employed to analyze large biological datasets, to predict AMP and antibiofilm activity, and to generate novel peptide sequences with desired properties. These approaches offer a more efficient and cost‐effective strategy for identifying potential antimicrobial peptides, reducing the reliance on traditional experimental methods. As research in this field progresses, computationally designed AMPs could play an important role in addressing the global challenge of antimicrobial resistance and developing next‐generation treatments.

ML [20] is a subset of artificial intelligence (AI) [21] that enables systems to learn from data, identify patterns, and make decisions without explicit programming. ML algorithms improve their performance over time by analyzing historical data and adjusting their model accordingly. ML is broadly classified into supervised learning [22] and unsupervised learning [23] and is widely used in applications such as fraud detection [24], recommendation systems [25], and biomarker prediction [26], driving automation [27] across different industries. In 2018, Yoshida et al. utilized machine learning combined with a generic algorithm and designed an evolutionary method for novel peptide generation. Without prior knowledge or databases, their method significantly enhanced antimicrobial activity, achieving a 162‐fold improvement within three iterations, demonstrating that ML predictions efficiently navigate the vast peptide sequence space, reducing the need for extensive chemical synthesis and accelerating AMP discovery [28].

DL is a specialized branch of machine learning that utilizes artificial neural networks with multiple hidden layers to process complex data [29]. DL architecture, such as recurrent neural networks (RNNs) [30], is designed to mimic human cognitive functions, allowing them to learn hierarchical representations of data. Long short‐term memory (LTSM) networks, a subtype of RNN neural network, are designed to effectively learn and process sequential data, which makes them promising for peptide design. Research has been conducted using LTSM models to generate new peptide sequences [31, 32, 33]. LSTM overcome the limitation due to the vanishing gradient [34] problem produced by RNN, by incorporating a unique gating mechanism that can regulate information flow, allowing them to maintain long‐range dependencies and selectively update, ignore, or pass on the information as needed. In 2022, Li et al. [35] utilized multibranch CNNs, where multiple parallel CNN structures were implemented, each followed by a semantic learning component to better capture peptide features. Additionally, they replace the gated recurrent unit (GRU) of RNNs with a bidirectional long short‐term memory (Bi‐LSTM) network, which improves the model's ability to capture contextual dependencies in peptide sequences. These designs were used here to develop more effective representations of bioactive peptides, ultimately achieving high prediction accuracy. Despite the discovery of numerous antimicrobial peptides (AMPs), the identification of those with both potent antimicrobial and antibiofilm activity remains a significant challenge. Most existing computational AMP design tools focus solely on antimicrobial efficacy against planktonic bacteria, neglecting biofilm inhibition, which is critical for treating chronic and device‐associated infections. Additionally, few studies have combined numerical feed‐forward neural network predictions with sequential deep learning architectures to improve multiobjective peptide design. Thus, there is a clear need for a predictive framework capable of identifying peptides that exhibit dual functionality and generalize across diverse sequence spaces. This study addresses the need by introducing a novel DeepQSAR approach that integrates feed‐forward neural network derived biofilm inhibition signals with a recurrent neural network for classification, enabling accurate prediction and design of peptides with enhanced multifunctional antimicrobial properties. Moreover, we integrate molecular dynamics (MD) simulations with biological assays to unravel how these peptides achieve their effects.

## 2 Methods

### 2.1 In‐House Training Set

Peptide arrays on cellulose membranes were made by Kinexus Bioinformatics Corporation (Vancouver, BC, Canada) and were obtained as cleaved free peptide associated with the punched‐out spot of the cellulose membrane. Stock solutions of the peptide array samples were prepared by dissolving the free peptide in 200 µL of endotoxin‐free sterile water, incubating at room temperature for ~ 1 h, and then transferring the resulting peptide solution to a sterile microfuge tube for subsequent antimicrobial activity assays. The concentration of the stock peptide array samples was determined by measuring the absorbance at 280 nm of the solution (in duplicate) on a BioTek Epoch Microplate Spectrophotometer using the Take3TM Multi‐Volume Plate. A theoretical extinction coefficient of each peptide was determined based on the number of Trp and Try residues1 in the peptide sequence according to the formula: (#Trp) × 5500 M^−^^1^ cm^−^^1^ + (#Tyr) × 1490 M^−^^1^ cm^−^^1^, and the concentration of the peptide in solution was calculated according to the Beer–Lambert law. Any Cys residues within the peptide samples were assumed not to be present in a disulfide bond, so their contribution to the extinction coefficient at 280 nm was ignored. Stock concentrations of peptides lacking a Trp and Tyr residue were assigned the average concentration of all the samples for which a concentration could be determined, which was 393 µM.

This dataset comprised approximately 700 unique peptides, each tested for antibiofilm IC_50_ values, resulting in 3,000 series arrays of numerical data. The IC_50_ values quantify the inhibitory concentration required to reduce biofilm formation by 50%, serving as a key indicator of peptide efficacy.

### 2.2 Antimicrobial Activity

Peptides were synthesized on peptide arrays and tested as described previously. Briefly, the antimicrobial activity and biofilm inhibition activity of the peptide array samples were assessed against methicillin‐resistant *Staphylococcus aureus* (MRSA) [36]. Briefly, twofold serial dilutions of the peptide array sample (10 μL final volume) were prepared in 96‐well plates and then 90 μL of a 1:100 dilution of an overnight culture of MRSA diluted in 10% tryptic soy broth supplemented with 0.1% glucose was added to each well and the plates were incubated overnight at 37°C. The following day, bacterial growth was quantified by measuring the optical density at 600 nm (OD_600_) of each sample well while the amount of biofilm was quantified by rinsing the wells of the plate with distilled water and staining the adhered biomass with 0.1% crystal violet solution. The amount of adhered biomass was quantified by recording the absorbance at 595 nm (A_595_). Dose–response curves were drawn for each peptide based on the bacterial growth (OD_600_) or biofilm inhibition (A_595_) and normalizing the percent of peptide inhibition relative to untreated bacteria (100% growth) or sterile media (0% growth). The concentration of peptide required to inhibit 50% of bacterial or biofilm growth (IC_50_) was calculated by nonlinear regression fit of the dose–response curves using the N‐Parameter Logistic Regression (nplr) package.

### 2.3 Public Training Datasets

Three publicly available antimicrobial peptide (AMP) datasets were incorporated.

1. DRAMP (Database of Antimicrobial Peptides) [37] contains 22,259 peptide entries.
2. AI4AMP [38] is a balanced dataset consisting of 10,716 positive AMPs and 10,718 negative AMPs.
3. DBAASP [39] (Database of Antimicrobial Activity and Structure of Peptides) contains 19,751 active AMPs with experimentally validated antimicrobial properties.

The combination of these datasets ensured the model learnt from a diverse range of peptide sequences for the classification model, enabling better generalization across different peptide properties and bioactivity profiles.

### 2.4 One‐Hot Encoding of Peptide Sequences

**FIGURE 1:**
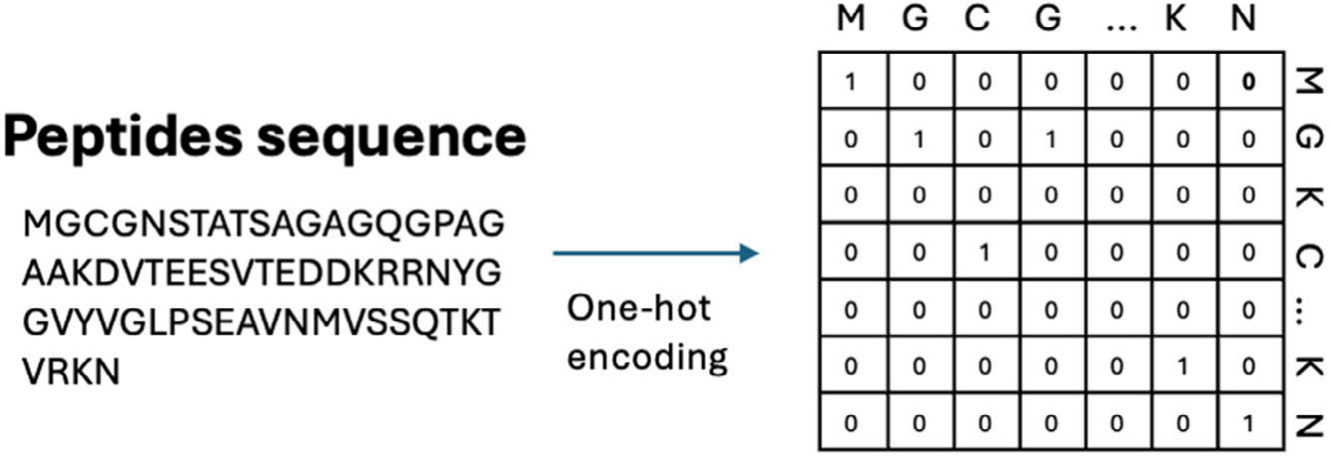
One‐hot encoding of peptides sequences: Each amino acid is represented as a unique binary vector while preserving sequence order.

One‐hot encoding is a technique used to convert categorical data, such as peptide sequences, into a format that can be efficiently processed by machine learning models [40, 41]. In the context of peptide sequences, one‐hot encoding transforms each amino acid into a unique binary vector, allowing machine learning algorithms to process the sequence data while preserving the order of the amino acids. We encoded 20 standard amino acids to unique binary vectors, each of length 20 (Figure 1). These vectors are embedded where “1” at the position corresponds to the amino acid in the set, and “0”s in all other positions. The entire peptide sequence is encoded as a matrix, where each row corresponds to the one‐hot vector representation of the amino acids in the peptide. One of the key features of one‐hot encoding is that it maintains the order of amino acids in the sequence.

### 2.5 Deep QSAR Modeling

#### 2.5.1 Feed‐Forward Neural Network

We began by constructing a feed‐forward neural network model (Model 1) using the in‐house dataset, with IC_50_ values on antibiofilm activities. The feed‐forward neural network model was trained on one‐hot encoded input vectors, focusing on numeric predictions of biofilm inhibition efficacy. To ensure a robust baseline, the model learned the quantitative relationships between the sequence features of peptides and their corresponding antibiofilm activity. The dataset used for this model consisted of peptides with known biofilm inhibitory properties, and the model performance was evaluated by comparing its predicted IC_50_ values to experimentally determined values. This feed‐forward neural network model provided a solid foundation for further development and enhanced our understanding of the quantitative aspects of biofilm inhibition.

#### 2.5.2 Recurrent Neural Network (RNN)

To enhance the performance of Model 2, we transferred the learned weights from Model 1 into the RNN architecture. This transfer of weights allowed the RNN to leverage the numerical insights and patterns learned from the feed‐forward neural network model, facilitating the classification task. The RNN was then trained on a labeled dataset of peptides, each with a known antimicrobial property. These labels allowed the RNN to focus on learning sequence‐specific patterns that contribute to antimicrobial activity, while also benefiting from the feed‐forward neural network model's predictions of biofilm inhibition.

#### 2.5.3 Combined Approach Integrating Both Model 1 and Model 2

**FIGURE 2:**
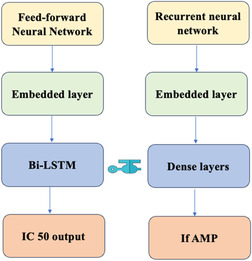
Model architecture. Model 1 is a feed‐forward neural network model built on IC_50_ values on antibiofilm activities. Model 2 is a classification model on antimicrobial property with weights learnt from model 1.

The integration of Model 1 and Model 2 was carried out by concatenating the output of the feed‐forward neural network model (Model 1) with the RNN architecture of Model 2 (Figure 2). This combined approach provided the RNN with valuable numerical insights from Model 1, which were incorporated as additional features to assist in classification. By using the feed‐forward neural network model's predictions as input, the RNN was able to focus not only on sequence‐based patterns but also on the broader quantitative relationships between peptide structure and biofilm inhibition, which were learned from the feed‐forward neural network model.

The transfer of weights from Model 1 to Model 2 ensured a smoother transition of learned features and improved the RNN's performance. The RNN's ability to predict antimicrobial activity was enhanced by the foundation provided by the feed‐forward neural network model, creating a more effective classifier for identifying antimicrobial peptides.

This integrated approach, combining the quantitative insights of the feed‐forward neural network model with the sequential learning ability of the RNN, allowed for more accurate predictions of peptide efficacy, especially in terms of both biofilm inhibition and conventional antimicrobial properties.

#### 2.5.4 Peptide Clustering

We applied a clustering method for narrowing down the number of peptides to be synthesized. To keep the diversity of peptide selection, the clustering method unitized the pairwise peptides sequences. We first calculated peptide similarity through global alignment, by aligning every pair of sequences from the beginning to end of each peptide. Then, a symmetric similarity matrix was generated as the input to the hierarchical clustering approach (AgglomerativeClustering from sklearn.cluster), which uses Euclidean distance to complete the linkage between clusters. The highest prediction score peptide from our combined QSAR model was selected from each cluster, ensuring broad representation from all regions of peptide sequence space.

#### 2.5.5 Molecular Dynamics

Using GROMACS [42], we simulated 43 designed 12‐mer peptides (categorized as antiplanktonic, dual, or antibiofilm), the positive control IDR‐1018, a composition‐matched scrambled IDR‐1018, and 14 length‐matched DRAMP “inactive” 12‐mers (lacking Lys/arg) using coarse‐grained MARTINI 3 [43]. For each peptide, we built three membrane systems—Gram‐positive (GP; POPG:cardiolipin 3:1), Gram‐negative inner membrane (GN; POPE:POPG:cardiolipin 6:2:1), and mammalian control (MAM; POPC)—with INSANE R package in ~ 15 × 15 × 35 nm^3^ boxes at 0.15 M NaCl. Initial α‐helical peptide models were prepared at pH 7 and converted with martinize2 (structure‐biased; elastic network ‐ef 500, ‐el 0.41, ‐eu 0.9) [44]. Each system underwent steepest‐descent and conjugate‐gradient minimizations, NVT equilibration for 200 ps at 323 K (V‐rescale; restraints on PO_4_ headgroups), then 500 ps NPT at 1 bar (semi‐isotropic). Production trajectories were run for 50,000,000 steps (dt = 20 fs; 1 μs) per replica with three replicas per peptide per membrane, using MARTINI‐recommended Verlet neighbor lists, reaction‐field electrostatics (*ε*_r_ = 15; 1.1–1.2 nm cutoffs), and semi‐isotropic pressure coupling; jobs were checkpointed and resumed until nsteps completed.

Analyses were performed with MDAnalysis [45]. At each saved frame, we computed peptide, Lys/arg, membrane, and phosphate (PO_4_) centers of mass; peptide‐membrane, Lys/arg‐membrane (primary cationic‐engagement metric), and peptide‐PO_4_ contact counts using a 0.5 nm cutoff; peptide tilt, RMSD/RMSF; and axial depth defined as z(COM_peptide) – z(midplane) where the midplane was obtained from the two PO_4_ layers. Replica time series were uniformly resampled to 1000 frames and averaged to peptide‐level traces without replicate dropping. GP trajectories informed planktonic metrics, GN trajectories informed antibiofilm metrics, dual peptides were analyzed in both backgrounds (reported as GP for kinetics and selectivity; GN for residency where indicated), and POPC served solely as the baseline for Δ versus MAM. General contact was defined as any interaction meeting the 5‐angstrom threshold. “Engaged” frames were defined as those with Lys/arg contacts ≥ 2. Bacteria‐over‐host selectivity was summarized as delta Δ‐contacts, computed per peptide as mean Lys/arg contacts in GP or GN minus MAM. SAR used Spearman correlations between late‐window depth (last 20% of frames) and log_10_(IC_50_); log‐linear fits were reported for finite pairs. Only category‐mean time‐series in the figure were smoothed (Gaussian *σ* = 5 frames); all statistics used unsmoothed data.

## 3 Results

It should be noted that research on simultaneous prediction of AMP multiple endpoints such as antimicrobial and antibiofilm activities of peptides remains limited, necessitating the development of a more comprehensive predictive model. To address this gap, we designed a combined Deep QSAR model that integrates feed‐forward neural network predictions with a sequence‐based LSTM model using transfer learning. This combined QSAR model enhanced the accuracy and efficiency of antimicrobial peptide (AMP) discovery by integrating advanced ML techniques. Our approach involved two key models: a feed‐forward neural network model (Model 1) and a recurrent neural network (RNN) model (Model 2). Model 1 was designed to predict antibiofilm IC_50_ values of peptides using an in‐house dataset with one‐hot encoded peptide sequences, enabling numerical predictions of biofilm inhibition efficacy. This feed‐forward neural network model served as a foundational step, capturing quantitative relationships between peptide sequences and their antibiofilm activity.

Building upon the insights gained from Model 1, we constructed Model 2, an RNN‐based binary LSTM classification model, to determine whether a given unknown peptide is likely to exhibit antimicrobial properties. To improve the RNN's predictive capability, we transferred the learned weights from Model 1, allowing it to leverage the peptide characteristics derived from the feed‐forward neural network model. The integration process involved concatenating the output of Model 1 with the RNN architecture of Model 2, enabling the classification model to utilize numerical insights, while focusing on sequence‐based patterns essential for antimicrobial prediction. This combined approach facilitated a more effective learning process, enhancing model performance and improving the accuracy of AMP identification. Through the use of this method, we demonstrated the potential of transfer learning and sequence‐based features in advancing computational peptide discovery. To further validate our approach, we conducted a large‐scale screening process followed by wet lab experiments.

In particular, we extracted 20,417 reviewed human protein sequences from Uniprot [46], filtered out proteins shorter than 100 amino acids, and systematically divided them into predicted peptide fragments, each 12 amino acids in length, using a sliding window approach. Using the developed combined QSAR model, we then screened approximately 50,000 peptides to predict their antimicrobial and antibiofilm activities. Based on the model's positive predictions, we clustered the peptides into 100 unique clusters to ensure selection diversity, then selected top‐ranked peptides in each cluster for chemical synthesis and subsequent biological testing. 98 out of top peptides in each of 100 distinct clusters were synthesized and experimentally evaluated for both biofilm inhibition and planktonic antimicrobial activity using peptide arrays. Their effectiveness was assessed compared to a known antibiofilm and antimicrobial peptide IDR‐1018, used as a positive control. These experimental results provided strong validation of our computational approach, demonstrating that our combined model can effectively predict bioactive peptides with multifunctional antimicrobial properties.

### 3.1 Model Performance

**FIGURE 3:**
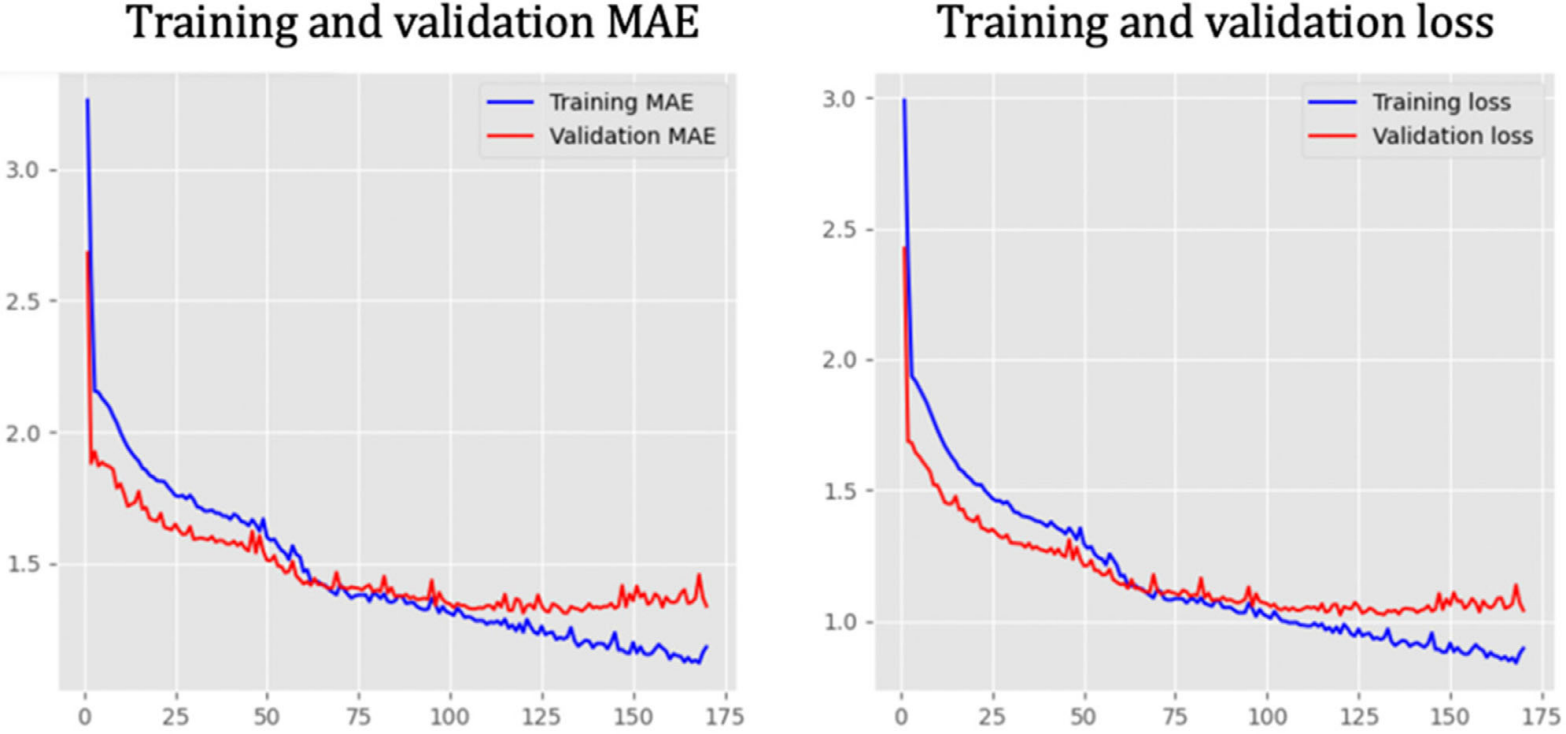
Feed‐forward neural network performance on training and validation mean absolute error (MAE) and loss for Model 1.

**FIGURE 4:**
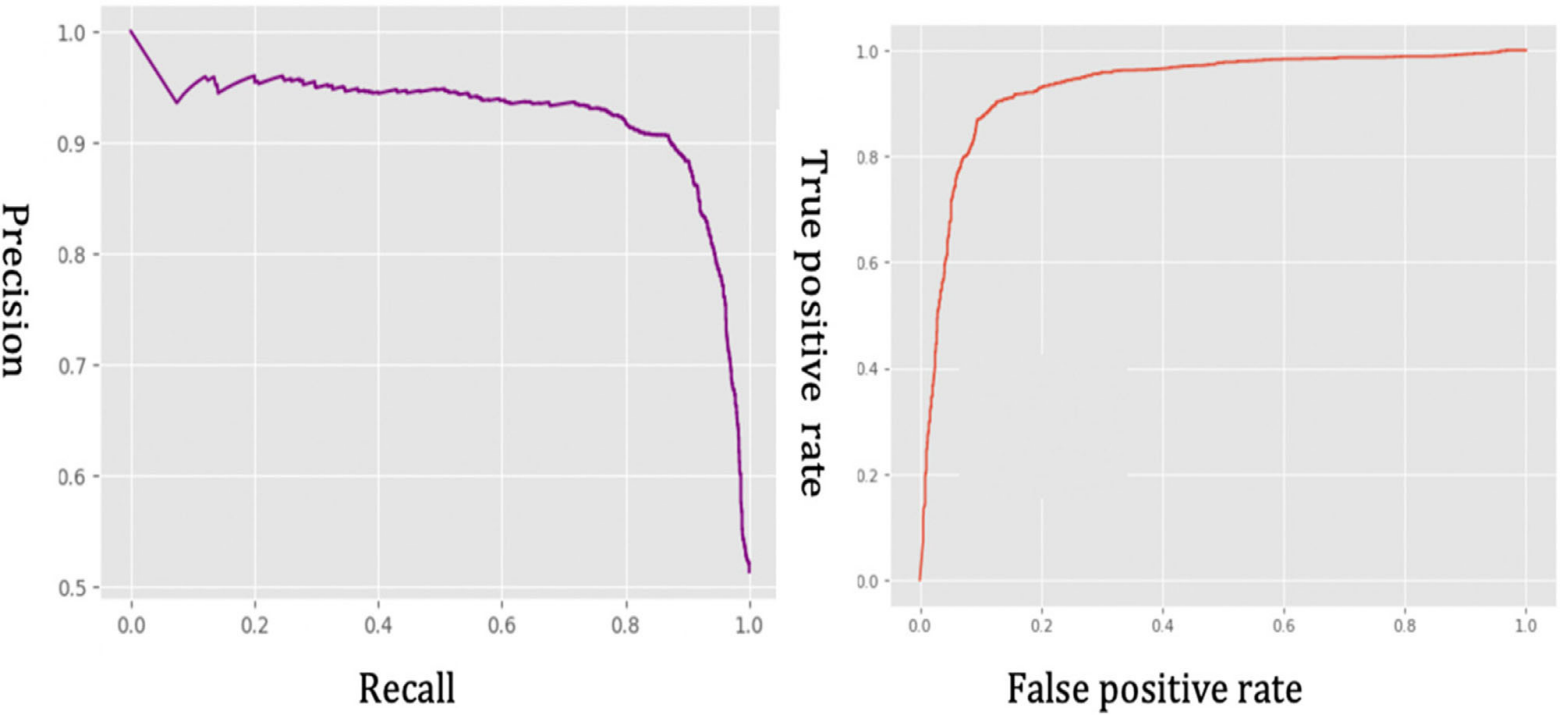
Precision‐recall (left) and ROC (right) curves for Model 2.

The developed model demonstrated strong performance throughout the training and validation phases, as reflected in key evaluation metrics such as AUC, F1, precision, and recall scores. Thus, the training and validation mean absolute error (MAE) [47] and loss curves indicate stable learning progress, with decreasing trends over time, suggesting that both Model 1 and Model 2 successfully captured meaningful patterns in the data (Figure 3). The precision–recall curve highlighted the model's high precision across different recall levels, while the receiver operating characteristic (ROC) curve showed a high true positive rate with a low false positive rate, reinforcing the reliability of our predictions (Figure 4).

To quantitatively assess the performance of the combined model, we evaluated precision, recall, and F1 score. The combined model achieved a precision of 0.90 for positive samples on active antimicrobial peptides and 0.87 for negative non‐antimicrobial peptides samples, with recall values of 0.88 and 0.89, respectively. The F1 scores of 0.89 for both classes indicated a well‐balanced performance in predicting antimicrobial peptides. These results indicated that the developed combined approach was highly effective, outperforming most existing AMP prediction models and demonstrating strong generalization in predicting unseen peptide sequences.

### 3.2 Peptides Synthesis and Validation

The antimicrobial activity and biofilm inhibition activity of peptide array synthesized samples were assessed against the important AMR human pathogen methicillin‐resistant *Staphylococcus aureus* (MRSA) [36]. Based on model predictions and clustering, we selected the top 100 peptides for synthesis and experimental validation. Their antimicrobial efficacy was tested against both biofilm and planktonic bacterial populations, with activity compared to a known antimicrobial peptide (AMP), IDR‐1018 [6], which served as a control. The results provided insights into the relative potency of these peptides in inhibiting bacterial growth and biofilm.

Among tested peptides, several exhibited superior antibiofilm and antiplanktonic activities compared to the control, as measured by IC50 values (µM). To enable a fair comparison between the peptides used for model training and the newly predicted peptides, we focused on strongly active peptides (IC50 values < 4 µM).

**FIGURE 5:**
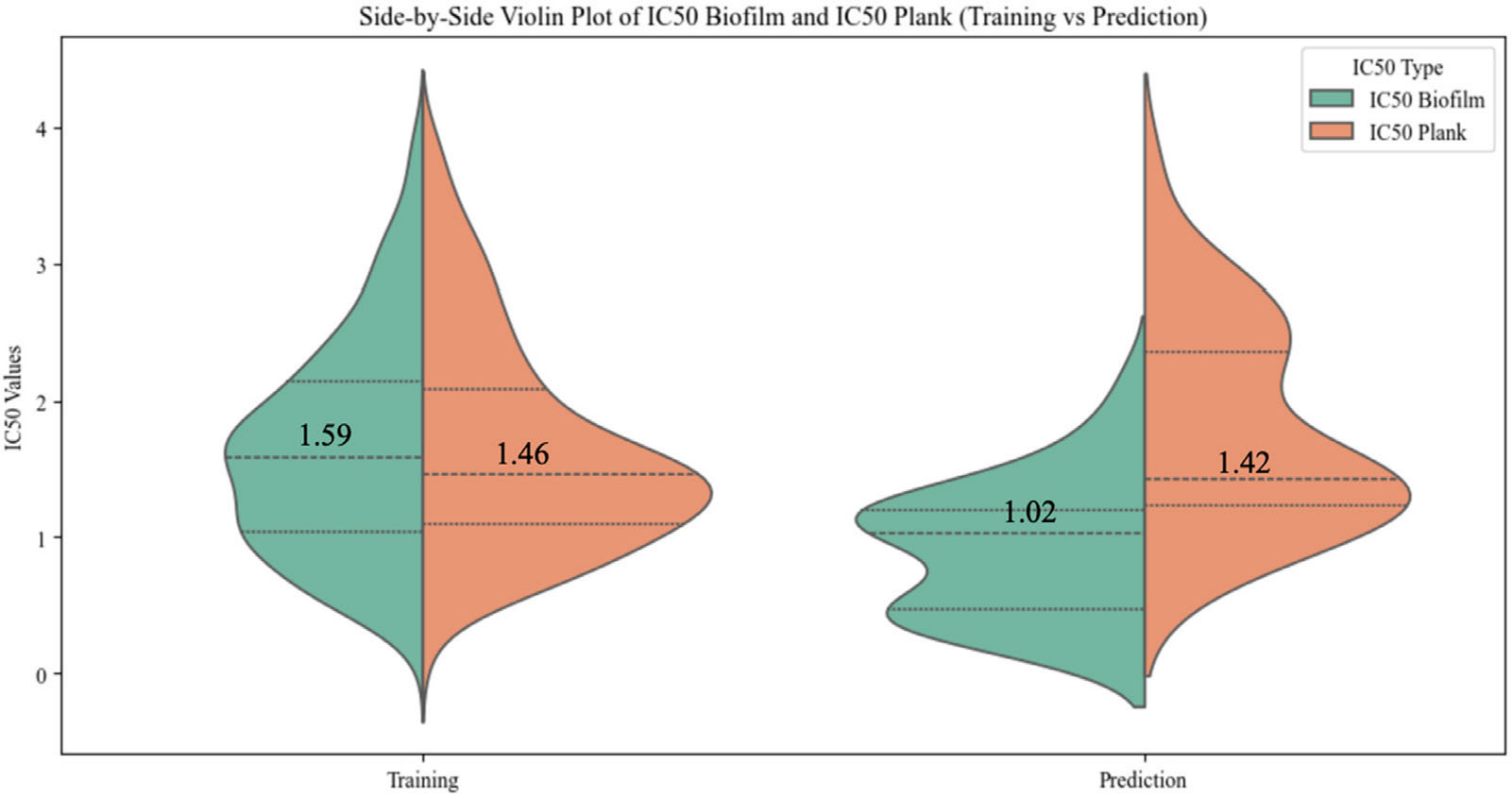
IC_50_ values comparison on antibiofilm and antimicrobial activities between training samples used in Model 1 and synthesized novel peptides. Median values are shown on the charts.

Overall, we compared the IC_50_ values vs. biofilms and planktonic bacteria between the peptides used in model training and the predicted novel peptides. The data for both sets were visualized using side‐by‐side violin plots (Figure 5), which illustrate the distribution of IC_50_ values for each group. The median IC_50_ values for these peptides in both antibiofilm and antimicrobial assays were consistently lower than those for the training peptides, providing strong evidence that the model can identify novel peptides with greater antibiofilm (and antimicrobial) activity.

**FIGURE 6:**
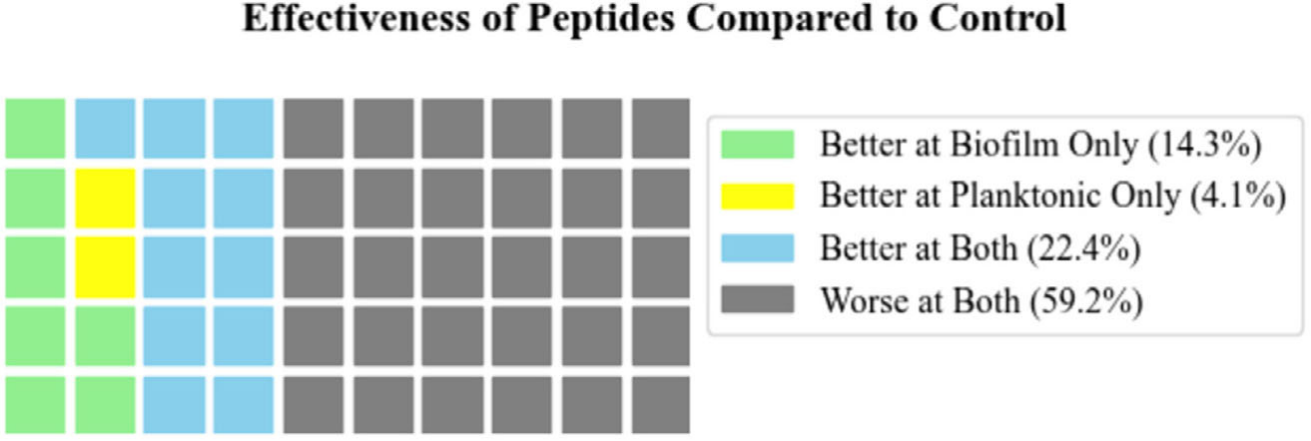
Effectiveness of synthesized peptides.

**FIGURE 7:**
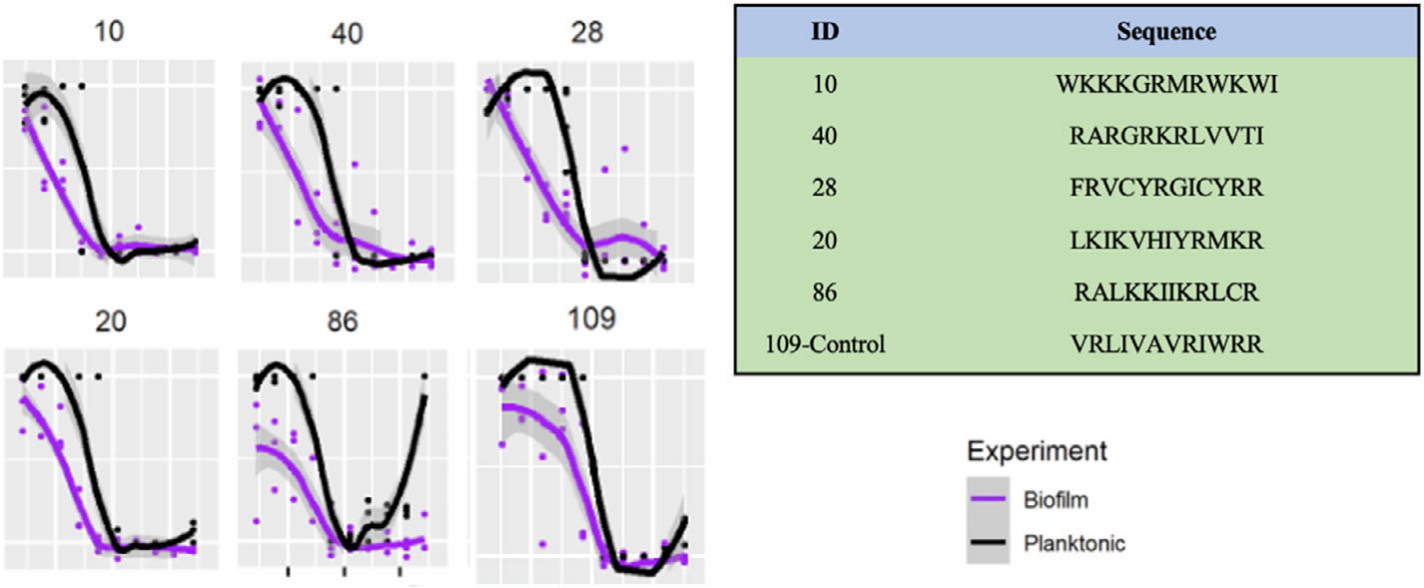
Array peptide biofilm vs. MRSA.

Specifically, 36 peptides demonstrated more effective antibiofilm activity than the control peptide IDR‐1018, 26 peptides exhibited stronger antiplank activity, and 22 peptides outperformed the control for both activities (Figure 6). The top 10 peptides for antibiofilm and antimicrobial activity are listed in Tables 1 and 2. Generally, peptides performed better against biofilms, a top target for such peptides and one for which conventional agents are poorly available. Notably, 5 peptides were common to both top 10 lists, highlighting their exceptional efficacy in antibiofilm and antimicrobial performance (Table 3, Figure 7).

**TABLE 1:** Top 10 effective antibiofilm peptides with IC_50_ value (in μM) on biofilms compared with the positive control peptide IDR‐1018 [6]. All peptides were amidated at the C‐terminus.

ID	Sequence	IC_50_ Biofilm
39	RGFVRLKKWFNI	0.23
10	WKKKGRMRWKWI	0.27
59	FRVCYRGICYRK	0.30
40	RARGRKRLVVTI	0.30
28	FRVCYRGICYRR	0.35
20	LKIKVHIYRMKR	0.35
86	RALKKIIKRLCR	0.38
50	KLVRRKGRLYVI	0.40
81	RVVRRKGRIYIL	0.44
9	LFKFLGKKVLKT	0.47
IDR‐1018	VRLIVAVRIWRR	1.42

**TABLE 2:** Top 10 effective antimicrobial peptides with IC_50_ value (in μM) for planktonic (medium‐based) organisms compared with the positive control IDR‐1018 [6]. All peptides were amidated at the C‐terminus.

ID	Sequence	IC_50_ antimicrobial
86	RALKKIIKRLCR	0.71
35	KGRIYVINKVQR	0.72
10	WKKKGRMRWKWI	0.74
62	RRRAKGRIRLIV	0.89
2	GRMRWKWIKKRI	1.03
20	LKIKVHIYRMKR	1.07
33	GLKSFARVLKKI	1.15
40	RARGRKRLVVTI	1.18
44	RLHGFLIRMRTR	1.19
28	FRVCYRGICYRR	1.21
IDR‐1018	VRLIVAVRIWRR	1.73

**TABLE 3:** Top 5 peptides extracted from top 10 rankings: dual superior antibiofilm and antimicrobial activity based on IC_50_ values compared to the positive control peptide IDR‐1018 [6]. All peptides were amidated at the C‐terminus.

ID	Sequence	IC_50_ Biofilm	IC_50_ Plank
10	WKKKGRMRWKWI	0.27	0.74
20	LKIKVHIYRMKR	0.35	1.07
28	FRVCYRGICYRR	0.35	1.21
40	RARGRKRLVVTI	0.30	1.18
86	RALKKIIKRLCR	0.38	0.70
IDR‐1018	VRLIVAVRIWRR	1.42	1.73

Notably, peptide RGFVRLKKWFNI (ID 39) demonstrated the strongest biofilm inhibition with an IC_50_ of 0.23 µM, significantly lower by nearly 6‐fold than the prominent antibiofilm peptide positive control, IDR‐1018 (1.417 µM). Additionally, it demonstrated improved planktonic activity (IC50 = 1.42 µM) relative to the control (1.73 µM). Other peptides, such as RALKKIIKRLCR (ID 86) and RARGRKRLVVTI (ID 40), also exhibited enhanced biofilm inhibition with IC_50_ values of 0.38 and 0.30 µM, respectively.

These findings highlight the effectiveness of our combined Deep QSAR model in identifying peptides with superior antibiofilm and antimicrobial properties, particularly in inhibiting biofilm formation, which is crucial for combating chronic bacterial infections, a major target of such peptides^41^ and one for which ML approaches are rare.

To evaluate the relative performance of our DeepQSAR model, we benchmarked its predictions against three widely used antimicrobial peptide (AMP) classifiers: Macrel, AI4AMP, and the DBAASP prediction tool. The comparison was conducted using the subset of 22 peptides from our designed set that exhibited experimentally validated superior antimicrobial and antibiofilm activity. Macrel [48] is an end‐to‐end pipeline specifically optimized for genome and metagenome mining. It uses a set of 22 features that comprises both local and global physicochemical descriptors such as charge and hydrophobicity to identify potential AMPs from short open reading frames. We screened superior 22 novel peptides from our model prediction using Marcrel, predictions placed the majority of these validated peptides within a narrow score range of 0.50–0.60, near its default decision threshold (Figure S2). This led to poor discrimination between active and inactive peptides and resulted in high false‐negative rates. AI4AMP [38] is a deep learning model that uses a novel physicochemical encoding scheme and convolutional neural networks to evaluate AMP potential. Trained on up‐to‐date AMP and non‐AMP datasets with a strong focus on external validation, AI4AMP reported high precision and generalizability in screening novel peptides. It performed well in qualitative terms, assigning high AMP‐probability scores to most of the validated sequences. However, when using the probability score assigned to IDR‐1018—the reference peptide in our study—as a classification threshold, the resulting confusion matrix yielded a precision and recall of approximately 0.50 (Figure S3). DBAASP's AMP predictor [39] relies on three membrane interaction relevant descriptors: hydrophobic moment, charge density, and membrane‐depth potential to distinguish AMP from non‐AMP sequences. This classifier produced a clear bias toward AMP predictions (15 predicted AMP vs. 7 predicted non‐AMP), demonstrating its tendency to overpredict activity in difficult borderline cases. This false‐positive skew highlights a key limitation of the current model and underscores the need for additional negative training examples to improve discrimination (Figure S4).

Overall, benchmark models demonstrate that existing AMP prediction tools exhibit limited predictive reliability for distinguishing highly active sequences in our experimental context. Our DeepQSAR framework was specifically optimized using both antimicrobial and antibiofilm activity data and successfully enriched for highly potent peptides, highlighting its enhanced practical utility in AMP discovery pipelines.

### 3.3 Peptide Synthesis and Validation

To evaluate the safety profiles of predicted peptides, we conducted hemolysis and PBMC cytotoxicity assays on three representative candidates (J20: LKIKVHIYRMKR, J28: FRVCYRGICYRR, J39: RGFVRLKKWFNI). These peptides were randomly selected from the top 22 peptides predicted by our classification model to outperform the reference peptide IDR‐1018 in antibiofilm activity. All three peptides were synthesized at > 95% purity and tested in standard in vitro assays. Hemolysis assays showed that each of the peptides exhibited negligible red blood cell lysis across a wide concentration range (1–256 μg/mL), with all three showing IC_50_ values > 250 μg/mL, indicating low membrane‐disruptive potential on human erythrocytes (Figure S1). PBMC cytotoxicity assays likewise revealed minimal toxicity. Peptides J28 and J39 demonstrated PBMC IC_50_ > 250 μg/mL, while J20 showed moderate cytotoxicity with IC_50_ = 166.1 μg/mL. These concentrations are well above the biofilm‐inhibitory concentrations (MBICs), which were 1–4 μg/mL for all three peptides (Table S1), suggesting favorable therapeutic indices. These results confirm that the model‐prioritized peptides not only exhibit potent antibiofilm activity but also retain low toxicity, supporting their suitability for further therapeutic development.

### 3.4 Molecular Dynamics Results

To connect sequence design to mechanism and the observed activity gains over IDR‐1018, we carried out triplicate 1 μs MARTINI‐3 simulations for every peptide in three bilayers aligned to the assays: an anionic Gram‐positive (GP) mimic for planktonic killing, a Gram‐negative (GN) inner‐membrane mimic for antibiofilm behavior, and a mammalian control (MAM, POPC) to quantify host selectivity. Within this framework, electrostatic capture by Lys/Arg—dominated by multidentate guanidinium–phosphate contacts—stabilizes carpet‐like interfacial states on anionic bacterial surfaces, whereas zwitterionic POPC disfavors such multivalent adhesion; shallow, persistent interfacial residence is therefore expected to correlate with lytic efficacy in GP, while GN antibiofilm activity can proceed after surface capture via intracellular pathways (such as (p)ppGpp interference), decoupling depth from potency [7].

**FIGURE 8:**
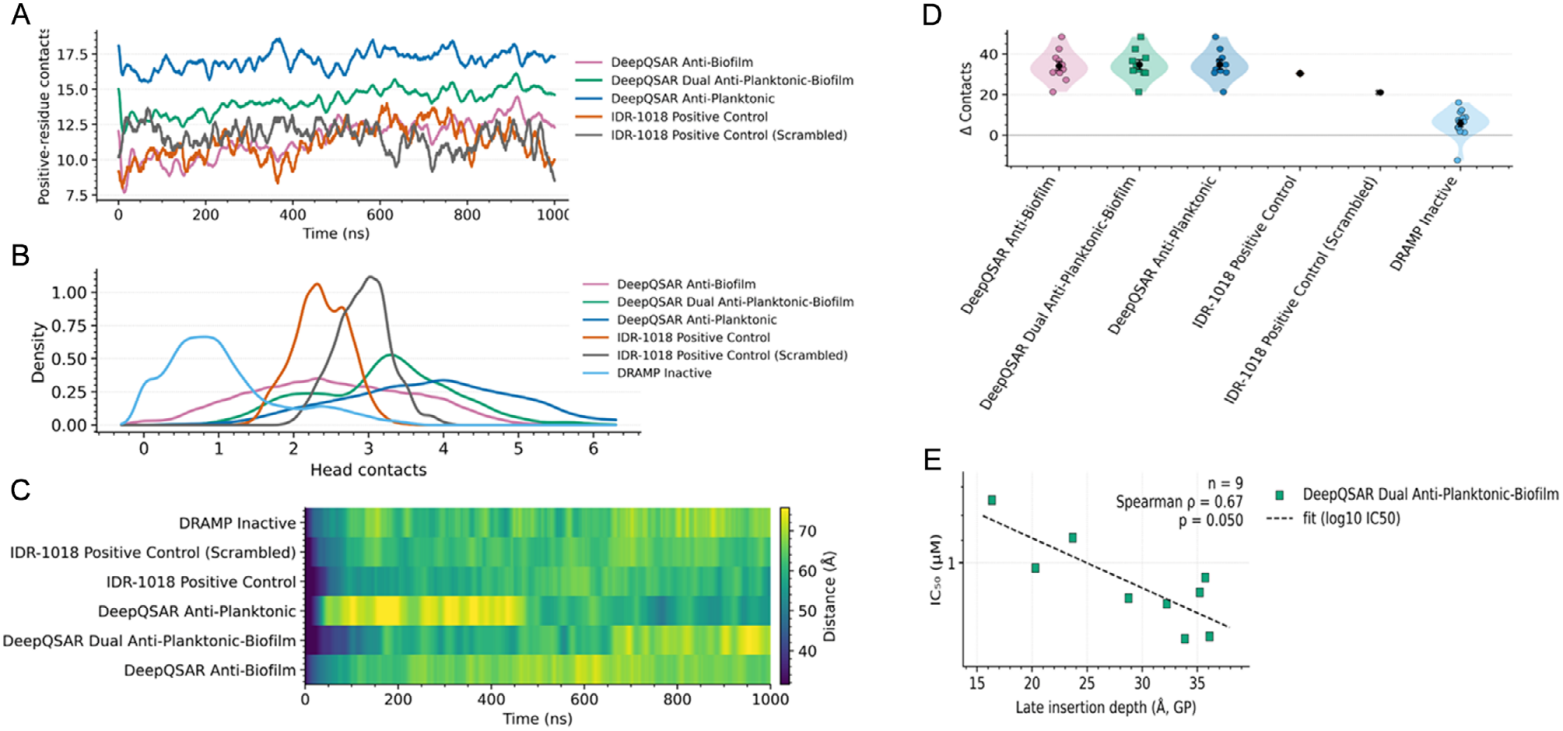
MD readouts of peptide–membrane engagement, residency, selectivity, and structure–activity.

Time‐resolved cationic engagement (5.0 Å cutoff) separated classes immediately (Figure 8A). Category‐mean Lys/Arg‐membrane contacts rose within tens of nanoseconds and remained high across 1 μs, with antiplanktonic peptides sustaining the largest burden in contact counts (mean ≈ 17.1; peak ≈ 18.6), dual peptides intermediate contact counts (mean ≈ 14.2; peak ≈ 16.1), and antibiofilm peptides lower on average contact counts (mean ≈ 11.5; peak ≈ 14.5) but showing the largest early‐to‐late increase in contact counts (Δ ≈ +3.3)—see the “Methods” section for defined engagement threshold. IDR‐1018 and the scrambled control remained in a similar range of contact counts (means ≈ 11.2–11.6). Consistent with their lack of Lys/Arg residues, DRAMP inactive 12‐mers showed no engaged frames at the predefined contact threshold. We then asked how peptides populate the head‐group layer. The PO_4_‐contact density \(Figure 8B) indicated higher head‐group engagement for DeepQSAR‐designed peptides than DRAMP inactive sequences, consistent with multivalent phosphate binding on anionic membranes and with established roles for Arg/Trp‐rich motifs in interfacial anchoring [49]. Across engaged frames (Lys/Arg contacts ≥2), the contact‐count distributions were shifted upward for antiplanktonic peptides (median 16 contact counts) compared to dual (median 13.3 contact counts) and antibiofilm (median 11.3 contact counts), with both controls centered at ~ 11–12 contacts. To complement instantaneous contacts with residency, we mapped peptide‐midplane distance over time as a COM‐distance heatmap aggregating category medians across replicas (Figure 8C; color scale ≈ 32–76 Å). Designed sets remained stably interfacial over 1 μs with near‐flat early‐to‐late drifts, whereas DRAMP remained more distant; IDR‐1018 showed sustained but less burdened interfacial residence, in line with its moderate but consistent binding signature [50].

Selectivity over the mammalian control was quantified as Δ‐contacts (total peptide‐membrane contacts at 5.0 Å) versus MAM (Figure 8D). DeepQSAR categories showed large positive shifts relative to POPC in both GP and GN (GP means ≈ +34 contacts; GN means ≈ +24–25). IDR‐1018 showed similarly positive Δ‐contacts (+30 in GP; +23 in GN), whereas the scrambled control was lower (+21 in GP; +16 in GN). DRAMP inactive sequences exhibited much smaller and more variable shifts (median 6.5; range −12.5 to 16 in GP; and median 2.7; range −15 to 11 in GN). These distributions account for the experimentally observed selectivity window—robust accumulation on bacterial membranes with minimal POPC engagement—and align with canonical electrostatics‐driven AMP selectivity [51]. Further corroborating the MD observables with the experimental assay outcome, in GP the late‐window insertion depth for the dual‐acting peptides correlated with MRSA planktonic IC50 (Spearman *ρ* = 0.67, *p* = 0.05) (Figure 8E) [52]. Consistent with antibiofilm modes that proceed after surface capture—most notably (p)ppGpp‐linked intracellular effects documented for the IDR‐1018 class—antibiofilm potency in GN is expected to be less tightly coupled to insertion depth.

Collectively, the microsecond simulations revealed a disproportionately disruptive profile for the DeepQSAR peptides compared to the baseline IDR‐1018 positive control and the DRAMP negative control with multivalent lys/arg adsorption driving rapid capture on anionic bacterial membranes, while the large engagement gaps over POPC provided practical optimization benchmark in the future.

## 4 Discussion

Here, we developed a Deep QSAR model capable of identification of novel enhanced antibiofilm and antimicrobial peptides. Through the filtering of human protein sequences in the UniProt database and selection of peptide fragments, we filtered 50,000 potential candidates and identified peptides with experimentally confirmed enhanced activity when compared to the positive control antibiofilm, antimicrobial peptide IDR‐1018 [6].

Overall, 37% of the top peptides reported higher biofilm inhibition than the excellent control peptide 1018, 27% exhibited greater antimicrobial (antibiotic) activities, and nearly 22% of peptides exhibited improved properties in both cf. IDR‐1018. These findings indicate the potential of our model in optimizing peptide discovery for antimicrobial applications.

One of the strongest findings of our approach is the integration of both sequence‐based screening and predictive modeling, which allowed us to successfully narrow down a high‐throughput dataset to a highly active subset of peptides. Unlike conventional AMP discovery methods requiring trial‐and‐error screening, our approach enhances the likelihood of identifying functional peptides with improved bioactivity.

A key insight from this work is the paramount importance of membrane interactions in underpinning the biological function of AMPs. By combining MD simulations with experimental assays, we demonstrated that strong, selective binding to bacterial membranes is a common denominator among the most effective peptides. This mirrors the mode of action of many host‐defense peptides, which first accumulate on bacterial surfaces and can then either form disruptive pores or cross into the cell [53]. The correlation we observed between depth of membrane insertion and antimicrobial potency suggests that peptides which can partially insert into the lipid bilayer are more likely to cause lethal membrane perturbations (such as pore formation, thinning, or the “carpet” mechanism of dissolving membranes). At the same time, those peptides, by virtue of being at the membrane interface, are well positioned to translocate into the bacterial cytosol. For antibiofilm function, intracellular entry is crucial for targeting the biofilm's molecular foundations (such as the stringent response via (p)ppGpp, as in IDR‐1018 [7] or other pathways that regulate biofilm maintenance). Thus, a peptide's ability to insert into membranes may serve a dual purpose: cause immediate damage at high concentration and enable cellular penetration at lower concentration—both outcomes contributing to biofilm eradication and bacterial killing.

Despite such promising results, further investigations are needed to establish the peptides’ mechanism of action, spectrum of activity and stability, toxicity, and activity in in vivo models. Additionally, structural analysis may also provide deeper insights into the interactions between these peptides and bacterial membranes, guiding further optimization.

Overall, our findings demonstrate the value of computational models in antimicrobial peptide discovery and uncover the potential for the development of novel therapeutics to combat biofilm‐associated and resistant infections. Future research should focus on refining the model and expanding its applications to identify peptides with even broader antimicrobial profiles.

## 5 Conclusions

This study demonstrates significant enhancement in identifying novel antibiofilm and antibiotic‐like peptides through a deep learning (DL) QSAR model that combines a recurrent neural network with feed‐forward neural network model. By utilizing the ability of the feed‐forward neural network model to determine quantitative relationships between peptide sequences and biofilm inhibition and combining this with the sequence‐based RNN in antimicrobial classification, this approach effectively predicted activity with high training accuracy to the level of 90% in 3‐fold cross‐validation. This robust predictive capability resulted in the identification of novel short cationic peptides with substantially improved antibiofilm and antimicrobial properties.

The peptides found by the constructed approach demonstrated more effective biofilm inhibition and antimicrobial activity compared to the training peptides, with stronger inhibition ability on average in antibiofilm and antimicrobial assays (Figure 3). The ability of the DL methodology to efficiently and simultaneously screen large peptide datasets for multiple therapeutic endpoints can enhance the precision and effectiveness of AMP discovery.

In conclusion, this work highlights the potential of AI‐based approaches to accelerate the identification of next‐generation therapeutics discovery, paving the way for novel strategies to combat resistant bacterial infections.

## Supporting Information

Additional supporting information can be found online in the Supporting Information section. **Supporting Fig. S1**: Hemolysis activity of peptides J20, J28, and J39 on human red blood cells across concentrations (1–256 μg/mL). **Supporting Fig. S2**: Performance of Macrel predictions on the 22 validated peptides, showing narrow score distribution (0.50–0.60) near the default threshold. **Supporting Fig. S3**: Performance of AI4AMP predictions on the 22 validated peptides, with probability scores benchmarked against reference peptide IDR‐1018. **Supporting Fig. S4**: Performance of DBAASP predictions on the 22 validated peptides based on hydrophobic moment, charge density, and membrane‐depth potential. **Supporting Table S1**: Cytotoxicity profiles of peptides J20, J28, and J39 against human PBMCs, compared with their minimum biofilm inhibitory concentrations (MBICs).

## Author Contributions

Jiaying You designed and applied the machine learning models, performed model screening, analyzed the results, and wrote the manuscript. Hazem Mslati performed and wrote the molecular dynamics (MD) simulation section. Evan F. Haney and Noushin Akhoundsadegh conducted the wet lab experiments and contributed to manuscript revisions. Robert E.W. Hancock supervised the performance and interpretation of the wet lab experiments, contributed to study design, and extensively edited the manuscript. Artem Cherkasov, as the corresponding author, helped design the modeling strategy and supervised the overall study.

## Funding

This study was supported by Natural Sciences and Engineering Research Council of Canada (grant RGPIN‐2024‐04153), Canada Foundation for Innovation (grant 36194), British Columbia Knowledge Development Fund (grant 36194), Canada Research Chairs Program (grant CRC‐2020‐00007).

## Conflicts of Interest

The authors declare no conflicts of interest.

## Supporting information

Supplementary Material

## Data Availability

All data used in this study, including in‐house antibiofilm activity data, model training datasets, and virtual screening results, are provided in machine‐readable format at a publicly accessible GitHub repository: https://github.com/chill‐bear/peptides. Python scripts for data preprocessing, model training, and figure generation are also available in the same repository.
