# Supplementary material for: The Use of DeepQSAR Models for the Discovery of Peptides With Enhanced Antimicrobial and Antibiofilm Potential
Source: Mol Inform. 2026 Apr 16;45(4):e70029. doi: 10.1002/minf.70029 (PMC13087548; doi:10.1002/minf.70029)
Supplement: Supplementary file 1 — Supplementary Material [file MINF-45-e70029-s001.pdf]

Supplementary Figures and Tables:

Figure S1: Hemolysis activity of peptides J20, J28, and J39 on human red blood cells across concentrations (1–256 μg/mL).

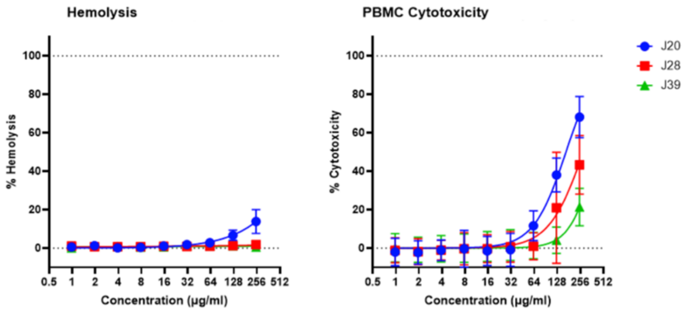

Figure S2: Performance of Macrel predictions on the 22 validated peptides, showing narrow score distribution (0.50–0.60) near the default threshold.

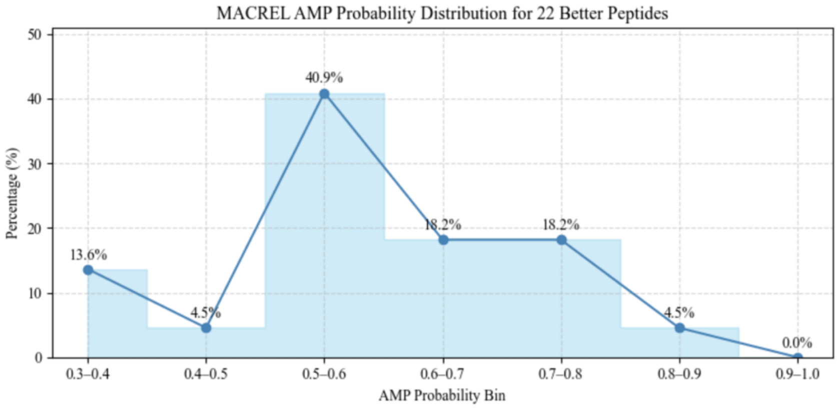

Figure S3: Performance of AI4AMP predictions on the 22 validated peptides, with probability scores benchmarked against reference peptide IDR-1018.

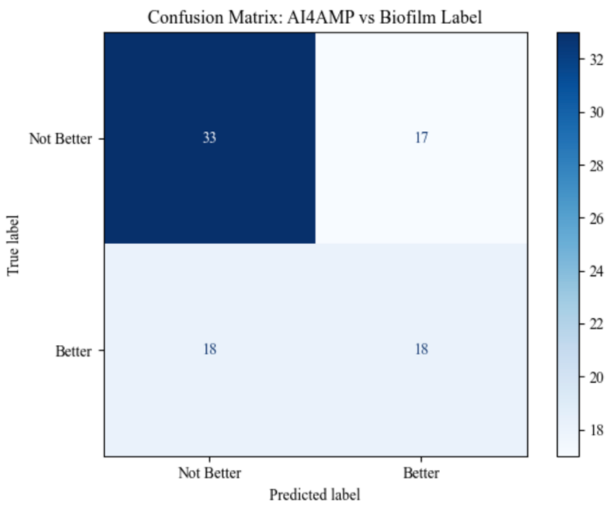

Figure S4: Performance of DBAASP predictions on the 22 validated peptides based on hydrophobic moment, charge density, and membrane-depth potential.

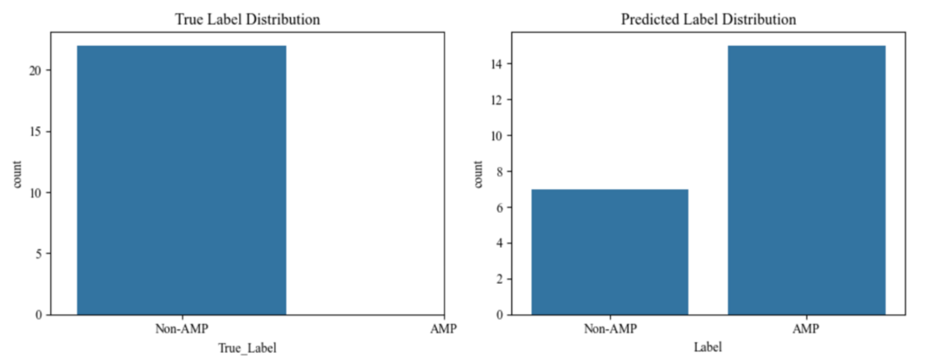

Table S1: Cytotoxicity profiles of peptides J20, J28, and J39 against human PBMCs, compared with their minimum biofilm inhibitory concentrations (MBICs).

Calculated IC_{50} Values for Hemolysis and PBMC Cytotoxicity

Peptide	Hemolysis IC_{50} (µg/ml)	PBMC Cytotoxicity IC_{50} (µg/ml)
J20	>250	166.1
J28	>250	>250
J39	>250	>250
